# Fish Oil Reduces Hepatic Injury by Maintaining Normal Intestinal Permeability and Microbiota in Chronic Ethanol-Fed Rats

**DOI:** 10.1155/2016/4694726

**Published:** 2016-04-10

**Authors:** Jiun-Rong Chen, Ya-Ling Chen, Hsiang-Chi Peng, Yu-An Lu, Hsiao-Li Chuang, Hsiao-Yun Chang, Hsiao-Yun Wang, Yu-Ju Su, Suh-Ching Yang

**Affiliations:** ^1^School of Nutrition and Health Sciences, Taipei Medical University, Taipei 110, Taiwan; ^2^Department of Nutrition and Health Sciences, Chang Gung University of Science and Technology, Taoyuan 333, Taiwan; ^3^National Applied Research Laboratories, National Laboratory Animal Center, Taipei 115, Taiwan

## Abstract

The aim of this study was to investigate the ameliorative effects of fish oil on hepatic injury in ethanol-fed rats based on the intestinal permeability and microbiota. Rats were assigned to 6 groups and fed either a control diet or an ethanol diet such as C (control), CF25 (control with 25% fish oil), CF57 (control with 57% fish oil), E (ethanol), EF25 (ethanol with 25% fish oil), and EF57 (ethanol with 57% fish oil) groups. Rats were sacrificed at the end of 8 weeks. Plasma aspartate aminotransferase (AST) and aminotransferase (ALT) activities, hepatic cytokines, and plasma endotoxin levels were significantly higher in the E group. In addition, hepatic histopathological analysis scores in the E group were significantly elevated. Rats in the E group also showed increased intestinal permeability and decreased numbers of fecal* Bifidobacterium*. However, plasma AST and ALT activities and hepatic cytokine levels were significantly lower in the EF25 and EF57 groups. Histological changes and intestinal permeability were also improved in the EF25 and EF57 groups. The fecal* Escherichia coli* numbers were significantly lower, but fecal* Bifidobacterium* numbers were significantly higher in the EF25 and EF57 groups.

## 1. Introduction

Chronic consumption of excessive ethanol leads to liver damage that may ultimately result in the development of alcoholic liver diseases (ALDs) including fatty liver, steatohepatitis, and cirrhosis [1]. Oxidative stress, lipid peroxidation, and inflammatory responses are all involved in the complex pathophysiological mechanisms of ALD [1]. There is an evolving concept that ethanol-induced dysbiosis disrupts the integrity of intestinal epithelium, resulting in intestinal inflammation and bacterial translocation [2]. Gut-derived endotoxin is a required cofactor, because an endotoxin-initiated hepatic necroinflammatory cascade leads to liver injury in ALD. Animal studies also showed that removal of the intestinal microflora with antibiotics prevents the occurrence of ALD [3–5]. Endotoxins are lipopolysaccharides (LPSs) derived from cell walls of gram-negative bacteria. Endotoxemia was found in ALD patients and gut leakage appears to be the cause of endotoxemia in ALD [6, 7]. Previous studies revealed that intestinal barrier hyperpermeability occurs only in alcoholics with ALD but not in those without liver disease [8]. Our previous study also indicated that synbiotics (combinations of probiotics and prebiotics) offers liver protection by improving the intestinal permeability and microbiota in rats with ethanol-induced liver injury [9]. Those findings mentioned above strongly suggest that intestinal barrier disruption induced by ethanol is the main mechanism of endotoxemia in ALD.

Dietary fish oil might be useful in preventing acute ethanol-induced fatty liver in animal models [10, 11]. Fish oil is rich in n-3 fatty acids, such as eicosapentaenoic acid (EPA) and docosahexaenoic acid (DHA), which decrease blood triglyceride (TG) concentrations in hypertriglyceridemia patients and show protective effects against fatty liver [11]. It was also indicated that EPA and DHA were particularly effective in supporting the intestinal barrier integrity by improving natural resistance and reducing the permeability of allergic and inflammatory mediators such as interleukin- (IL-) 4 and interferon- (IFN-) *γ* [12]. In addition, recent studies suggested that fish oil may influence contents of the gut microbiota, especially increasing beneficial bacteria such as* Lactobacillus* and* Bifidobacterium* [13, 14]. However, there is limited evidence from studies of the relationship between fish oil and ALD based on the viewpoint of the intestinal integrity and microflora.

Therefore, we hypothesized that fish oil may have protective effects against liver injury in ethanol-fed rats by improving the intestinal permeability and microbiota. This animal study of ethanol-induced liver injury was performed to investigate the proposed hypothesis.

## 2. Methods and Materials

### 2.1. Animals

Male Wistar rats which are 8 weeks old and weighing 280 g were used in this experiment (BioLasco Taiwan, Ilan, Taiwan). All rats were housed in individual cages in an animal room maintained at 22 ± 2°C and 50%~70% humidity with a 12 h light-dark cycle. Rats were allowed free access to a standard rodent diet (LabDiet ICN: AIN-76a Rodent Diet; PMI Nutrition International, St. Louis, MO, USA) and water during acclimation before the study. All procedures were approved by the Institutional Animal Care and Use Committee of Taipei Medical University.

### 2.2. Study Protocol

Aspartate transaminase (AST) and alanine transaminase (ALT) activities in plasma were analyzed before the experiment. Rats were assigned to groups based on the AST and ALT activities to make sure that the liver function did not differ among groups at the beginning of the experiment. Experimental models of ALD are commonly generated by feeding animals the Lieber-DeCarli liquid diet, in which fats in the diet are rich in monounsaturated fatty acids (MUFAs) and low in polyunsaturated fatty acids (PUFAs) [15]. Therefore, in this study, fish oil was used to substitute for part of the olive oil in the Lieber-DeCarli liquid diet. That is, 36 male Wistar rats were divided into six groups and fed either a control diet or an ethanol-containing diet, in which the fat composition of both diets was adjusted with 25% or 57% fish oil substituted for olive oil. The groups included C (control), CF25 (control with 25% fish oil), CF57 (control with 57% fish oil), E (ethanol), EF25 (ethanol with 25% fish oil), and EF57 (ethanol with 57% fish oil). MUFA/PUFA ratios of the diets without fish oil and with 25% and 57% fish oil substitutions were 0.4, 0.7, and 1.5, respectively (Table 2). Rats in the E group were fed an ethanol-containing liquid diet, while rats in the C group were pair-fed an isoenergetic diet without ethanol. The ethanol liquid diet in this study contained 35% energy as ethanol which was modified from Lieber and DeCarli's ethanol liquid diet [15], while paired-fed control rats (C, CF25, and CF57 groups) received an equal amount of calories as their ethanol-fed counterparts (E, EF25, and EF57) by substituting ethanol-derived calories with maltodextrin. The compositions are shown in Table 1 and fish oil (VIVA Omega-3*™*) was provided by VIVA Life Science (Costa, Mesa, CA, USA). One gram fish oil contains 250 mg EPA and 178.6 mg DHA. A pair-feeding procedure was conducted in this study; that is, the amount of the liquid diet consumed by rats of the E group was measured and then equal energy of the diet was provided to rats of the other five groups the next day.

At the 7th week, an intestinal permeability test and microbial culture of feces were carried out for all rats. All rats were sacrificed at the 8th week of the experiment. Blood samples were collected in heparin-containing tubes and centrifuged (1200 ×g for 15 min at 4°C) to obtain plasma samples. All plasma samples were stored at −80°C until being assayed. Liver tissues were rapidly excised. Parts of the liver tissues were fixed in 10% formaldehyde and embedded in paraffin for a histopathological analysis. Other liver tissues were stored at −80°C for further analysis.

### 2.3. Plasma Biochemical Indicators of Liver Function

AST and ALT activities were analyzed as biochemical markers of liver function using the SYNCHRON CX System Hitachi 7170 (Hitachi High-Technologies, Tokyo, Japan).

### 2.4. Hepatic Histopathological Analysis

Liver tissues fixed with formalin were processed with hematoxylin-eosin (H&E) staining and Masson's trichrome staining. The H&E stain was used to evaluate liver damage including hepatocyte fatty change, inflammatory response, degeneration, and necrosis. Masson's trichrome stain was used to assess collagenous fibers. A semiquantitative histological evaluation was performed by a pathologist blinded to the treatment groups to evaluate the severity of hepatic injuries. The grading ranged from 0 to 4 where 0 = absent, 1 = trace, 2 = mild, 3 = moderate, and 4 = severe.

### 2.5. Inflammatory Response

#### 2.5.1. Hepatic Cytokine Concentrations

Inflammatory cytokines including tumor necrosis factor- (TNF-) *α*, IL-1*β*, IL-6, and IL-10 levels were measured. Liver tissues (0.5 g) were homogenized in 1.5 mL ice-cold buffer (50 mM Tris (pH 7.2), 150 mM NaCl, and 1% Triton-X 100) plus 0.1% of a protease inhibitor. The homogenates were shaken on ice for 90 min and then the homogenates were centrifuged at 3000 ×g and 4°C for 15 min. The supernatant was analyzed with a DuoSet® rat TNF-*α* kit, a rat IL-1*β*/IL-1F2 kit, a rat IL-6 kit, and a rat IL-10 kit (R&D Systems, Minneapolis, MN, USA). Procedures followed the assay kit instructions. The optical density (OD) was read at 450 nm for all cytokines using a microplate reader (Molecular Devices, Sunnyvale, CA, USA).

### 2.6. Intestinal Permeability

#### 2.6.1. Plasma Endotoxin Level

Plasma endotoxin levels were measured using a limulus amebocyte lysate assay kit (Pyrochrome® Cape Cod, East Falmouth, MA, USA) and procedures followed the manufacturer's instructions. The OD was read at 405 nm using a microplate reader (Molecular Devices, Sunnyvale, CA, USA).

#### 2.6.2. Urinary Lactulose/Mannitol (L/M) Ratio

An oral sugar test was used to assess intestinal permeability [16]. Briefly, rats were intragastrically administered 2 mL of a sugar solution containing lactulose (100 mg/kg body weight (BW)), mannitol (6 mg/kg BW), and sucrose (200 mg/kg BW). Ten milliliter of lactated Ringer's solution was injected subcutaneously to rats before sugar administration to promote urine output. Then, rats were housed in metabolic cages individually and urine samples were collected for 5 h. Urinary sugar levels were analyzed by liquid chromatography/tandem mass spectrometry (LC-MS/MS, AB SCIEX QTRAP® 5500, Framingham, MA, USA). An increased urinary L/M ratio indicates that the intestinal permeability is elevated.

### 2.7. Microbiota Composition of Feces

In order to collect fresh feces, rats were anesthetized by ethyl ether inhalation and fecal samples were collected in an anaerobic dilution solution (4.5 g/L KH_2_PO_4_, 6 g/L Na_2_HPO_4_, 0.5 g/L L-cysteine: HCl, 2 g/L gelatin, and 1 mL/L Tween-20). Fecal samples were followed by 10-fold serial dilutions (10^−1^ to 10^−6^) to acquire different concentrations and 50 *μ*L of the solution was inoculated onto agar by the spread plate method for plate counts. Certain microorganisms were isolated from fecal samples using different isolation media. CDC anaerobe blood agar plates (A01-12, Creative Media Products, Taiwan) were used to detect the total aerobic bacterial flora. Endo agar plates (Difco*™* & BBL*™*, Becton, Dickinson and Company, Sparks, MD, USA) were used for detecting* E. coli*.* Lactobacillus* anaerobic MRS with bromocresol green (Difco*™* & BBL*™*, Becton, Dickinson and Company, Sparks, MD, USA) was used to detect* Lactobacillus*. Modified* Bifidobacterium* iodoacetate medium-25 (Difco*™* & BBL*™*, Becton, Dickinson and Company, Sparks, MD, USA) was used to detect* Bifidobacterium*. The number of colony forming units (CFU) of bacteria was quantified. Endo plates were incubated for 24 h at 37°C to count colonies of* E. coli*. CDC plates, modified MRS agar plates, and BIM-25 plates were incubated in anaerobic chambers for 48 h at 37°C to, respectively, count colonies of total aerobic bacterial flora,* Lactobacillus*, and* Bifidobacterium*.

### 2.8. Statistical Analysis

Data are presented as the means ± standard error of the mean (SEM). A two-way analysis of variance (ANOVA) followed by Fisher's test was used to determine statistical differences among groups using SAS software version 8.0 (SAS Institute, Cary, NC, USA). Statistical significance was assigned at the *p* < 0.05 level.

## 3. Results

### 3.1. Food Intake, Growth Performance, and Liver Weight

Average liquid dietary intakes of the C, CF25, CF57, E, EF25, and EF57 groups were 63 ± 2, 62 ± 2, 62 ± 2, 65 ± 2, 63 ± 2, and 64 ± 2 g/rat/day, respectively. Average ethanol consumption of rats in the E, EF25, and EF57 groups was 3.2 ± 0.1, 3.1 ± 0.1, and 3.2 ± 0.1 g/day.

There were no significant differences in initial BWs among groups. However, the final BWs of the E, EF25, and EF57 groups were significantly lower than that of the C group (*p* < 0.05). Relative liver weights of the E, EF25, and EF57 groups were significantly higher than that of the C group (Table 3).

### 3.2. Plasma Biochemical Indicators of Liver Function

AST and ALT activities of the E group were significantly higher than those of the C group (*p* < 0.05), while these two parameters in the EF25 and EF57 groups were significantly lower than those of the E group (*p* < 0.05) (Table 4).

### 3.3. Hepatic Histopathological Analysis

**Figure 1:**
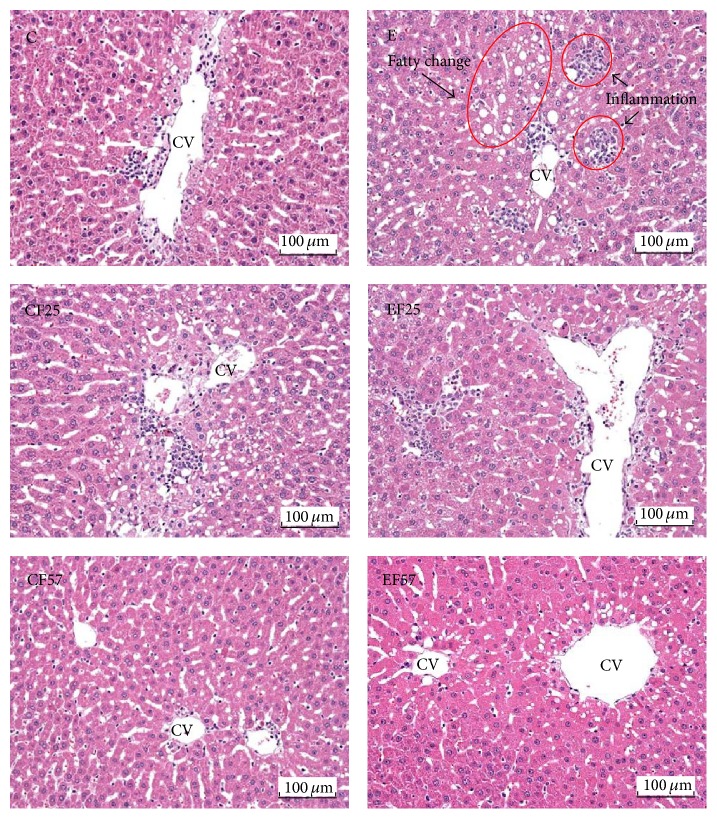
Representative photomicrographs of livers (H&E stain, magnification: ×200). C, control; CF25, control with 25% fish oil substituted for olive oil; CF57, control with 57% fish oil substituted for olive oil; E, ethanol; EF25, ethanol with 25% fish oil substituted for olive oil; EF57, ethanol with 57% fish oil substituted for olive oil. CV, central vein. Fatty change and inflammation (arrows) occurred in E group while there were few histopathological changes in the other groups.

**Figure 2:**
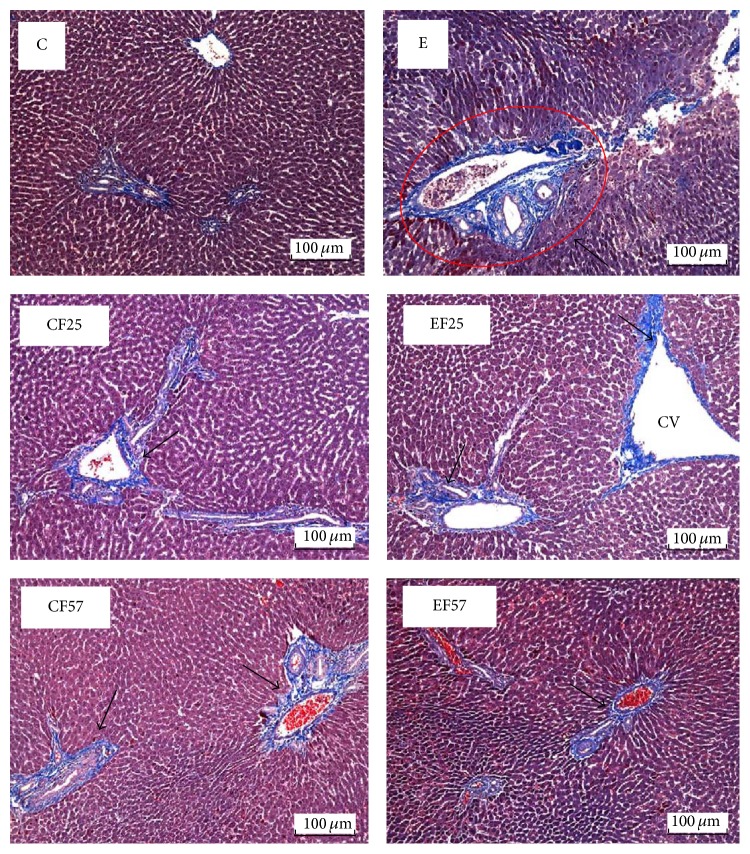
Representative photomicrographs of livers (Masson trichrome stain, magnification: ×200). C, control; CF25, control with 25% fish oil substituted for olive oil; CF57, control with 57% fish oil substituted for olive oil; E, ethanol; EF25, ethanol with 25% fish oil substituted for olive oil; EF57, ethanol with 57% fish oil substituted for olive oil. CV, central vein. Collagenous fibers (arrows) were stained in several biopsy specimens of E group; however, few collagenous fibers were found in the other groups.

Histopathological analysis scores were shown in Table 5. Hepatic fatty change, inflammation, and necrosis scores were significantly higher in the E group (*p* < 0.05), while scores of hepatic fatty change and inflammation were lower in the EF25 and EF57 groups than those in the E group (*p* < 0.05). On the other hand, hepatic degeneration and the necrosis score did not significantly differ between the EF25 and E groups, but the score of the EF57 group was significantly lower than that of the E group (*p* < 0.05). Moreover, the score of hepatic fibrosis was significantly higher in the E group compared to the C group. Conversely, only the fibrosis score of the EF57 group was significantly lower than that of the E group. The photomicrographs of the liver tissues showed that fatty change and inflammation were observed in the E group (Figure 1). Masson's trichrome staining showed that collagenous fibers were shown in several biopsy specimens of the E group; however, few collagenous fibers were found in the other groups (Figure 2).

### 3.4. Inflammatory Response

#### 3.4.1. Hepatic Cytokine Concentrations

Effects of fish oil on hepatic inflammatory cytokines in rats under chronic ethanol feeding are shown in Table 6. Chronic ethanol consumption (E group) led to a significant increase in hepatic TNF-*α*, IL-1*β*, IL-6, and IL-10 concentrations (*p* < 0.05). On the other hand, all of the hepatic cytokine levels measured in this study in the EF25 and EF75 groups were significantly lower compared to those of the E group (*p* < 0.05).

### 3.5. Intestinal Permeability

#### 3.5.1. Plasma Endotoxin Level

Plasma endotoxin levels are presented in Table 7. Those of rats in the E group showed significant elevation compared to those of rats in the C group (*p* < 0.05). There was no significant difference in plasma endotoxin levels between the E and EF25 groups, but levels in the EF57 group showed a dramatic decrease (*p* < 0.05).

#### 3.5.2. Urinary L/M Ratio

The E group showed the highest urinary L/M ratio among all groups (*p* < 0.05). However, ratios were significantly lower in the EF25 and EF57 groups than that of the E group (Table 8).

### 3.6. Microbiota Composition of Feces

The number of fecal* E. coli* in the E group showed an increasing trend compared to the C group. On the other hand, numbers of* E. coli* in the EF25 and EF57 groups were significantly lower than that in the E group (*p* < 0.05). Additionally, there were no significant differences in the number of anaerobes or* Lactobacillus* among all groups. However, the number of* Bifidobacterium* in the E group was significantly lower than that in the C group (*p* < 0.05). Conversely, the EF25 and EF57 groups presented significantly high numbers of* Bifidobacterium* compared to the E group (*p* < 0.05) (Table 9).

## 4. Discussion

Average ethanol consumption in the E, EF25, and EF57 groups was 3.1~3.2 g/day. According to a conversion of animal doses to the human equivalent based on the body surface area, this amount of ethanol intake in rats is equivalent to 73.6 g/day/person [17, 18], which can be considered as a heavy drinker in human [19, 20].

Although the caloric intake was identical among the all groups, the final BWs were found lower in the ethanol intake groups (Table 3). This result indicated that nutrients absorption and the efficiency of calorie utilization were affected when ethanol was administered for a long period of time [21]. Relative liver weights in the ethanol intake groups also significantly increased, which is consistent with our previous report [22]. The current study further indicated that substituting fish oil for olive oil in the liquid diet had no effect on BWs or hepatomegaly in chronic ethanol feeding rats.

In this study, plasma AST and ALT activities were measured to evaluate liver damage caused by chronic ethanol feeding. The enzyme activities in the E group were significantly higher than those in the C group after 8 weeks of ethanol feeding (Table 4). In addition, based on the histopathological analysis and photomicrographs of the livers (Table 5, Figure 1), fat accumulation was observed in the E group. This can be explained by enhancing free fatty acid mobilization from adipose tissues and increased biosynthesis of lipids, as previously suggested [22]. Furthermore, an inflammatory response also occurred in the E group (Table 5, Figure 1). These findings confirmed that ethanol consumption for 8 weeks led to liver injury in the present study. On the other hand, AST and ALT activities were significantly lower in rats fed the fish oil and ethanol-containing diet at the end of the study (Table 4). Similarly, according to results of the liver pathology (Table 5, Figure 1), we found that ethanol-induced fat accumulation and inflammation in the liver were alleviated by the dietary fish oil replacement. It was indicated that fish oil accelerates catabolism of very-low-density lipoprotein in the liver [23]. Therefore, we speculated that partial substitution of fish oil may have mitigated the liver damage by eliminating fat accumulation and then successively decreasing inflammation because of reduced fatty changes in the liver. On the other hand, we also found that only higher fish oil replacement (EF57 group) significantly reduced ethanol-induced fibrosis (Table 5, Figure 2). It is important to confirm the effects of fish oil on hepatic fibrosis-related factors, such as transforming growth factor- (TGF-) *β*1 in future studies.

Activation of the innate immune response by chronic ethanol exposure plays a major role in initiating and promoting alcoholic liver injury. Several steps, including intestinal bacterial LPS, increased gut permeability, endotoxemia, and Kupffer cell activation, trigger the inflammatory reaction [24]. Stimulation of Kupffer cells induces oxidative stress and produces proinflammatory cytokines, such as IL-1, IL-6, and TNF-*α*, that cause hepatocellular damage [25]. These cytokines lead to apoptosis and necrosis of hepatocytes and consequently result in liver injury [26, 27]. Anti-inflammatory cytokines are usually secreted with or after the production of proinflammatory cytokines, which in turn maintain homeostasis of immune response and protect the liver against injury [28]. IL-10 is an anti-inflammatory cytokine secreted by Kupffer cells and peripheral blood monocytes. In this study, we found that concomitant with the upregulated hepatic proinflammatory cytokines, IL-10 level was also elevated in the E group (Table 6). We speculated that IL-10 as an anti-inflammatory cytokine was secreted in response to the release of proinflammatory cytokines, because Kupffer cells were activated by chronic ethanol consumption. It might be concluded that there is an imbalance between pro- and anti-inflammatory mediators regulation during chronic ethanol exposure [29]. On the other hand, hepatic TNF-*α*, IL-1*β*, IL-6, and IL-10 concentrations in the EF25 and EF57 groups were significantly lower than those in group E (Table 6). Results showed that fish oil normalize hepatic pro- and anti-inflammatory cytokine secretions in rats under chronic ethanol abuse. This cytokine-lowering effect of fish oil might be one of the reasons explaining the minor inflammatory cell infiltration based on the hepatic histopathological analysis in the EF25 and EF57 groups (Table 5). A previous study indicated that fish oil could reduce ethanol-induced fatty liver and hepatic production of the inflammatory cytokines, IL-6 and TNF-*α*, consistent with the reduction of IL-6 having a protective effect against ethanol-induced hepatic steatosis [10]. This was confirmed by hundreds of references that the active ingredients in fish oil are EPA and DHA, which can competitively inhibit the secretion of proinflammatory interleukins, because EPA is converted into anti-inflammatory prostaglandins (PGs) of the PGE3 series [10].

In order to investigate relationships between intestinal integrity and ethanol-induced liver damage, plasma endotoxin levels and the intestinal permeability were measured. In this study, both the plasma endotoxin level and urinary L/M ratio were significantly higher in the E group (Tables 7 and 8). The mechanisms responsible for the influences of ethanol feeding on increasing intestinal permeability were proposed previously [30]. Firstly, chronic ethanol exposure promotes intestinal gram-negative bacteria growth which may result in accumulation of endotoxin. Secondly, intestinal bacteria and epithelial cells metabolize ethanol that may result in acetaldehyde accumulation, which in turn increased tyrosine phosphorylation of tight junction and adherens junction proteins and thus enhance the intestinal permeability to endotoxin [12]. Thirdly, ethanol induces the production of nitric oxide and superoxide that may decrease stable polymerized tubulin and increase disassembled tubulin levels and subsequently disrupt the intestinal barrier function. The increased intestinal permeability to peptidoglycan can initiate an inflammatory response in the liver [30].

The findings of this study revealed that chronic ethanol intake increased intestinal permeability, enhanced endotoxin translocation from intestines to the liver and systemic circulation, and triggered inflammatory response in the liver. However, ethanol exposure with 25% or 57% fish oil substituted for olive oil showed significantly lower plasma endotoxin levels (only in the EF57 group) and urinary L/M ratio (in both the EF25 and EF57 groups) (Tables 7 and 8). Previous study found that n-3 fatty acids changed the lipid environment in tight junction membrane microdomains, prevented the redistribution of tight junction proteins, and reduced the inflammation induced by TNF-*α* [31]. Therefore, we surmised that fish oil substitution for olive oil in rats fed the ethanol-containing liquid diet normalized the intestinal permeability and has lower plasma endotoxin level especially when higher fish oil was administered.

Normal intestine permeability and gut microbes are both important in maintaining intestinal health. It was indicated that species of* Bacteroides*,* Porphyromonas*,* Bifidobacterium*,* Lactobacillus*,* Clostridium*, and* E. coli* are the most frequent ones in the intestinal tract [28]. In this study, fecal microbes were analyzed to reflect gut microbes. We found that slightly higher numbers of* E. coli* and significantly lower numbers of* Bifidobacterium* in the E group were found (Table 9). This result was consistent with our previous report [9]. A clinical study also showed that alcoholic subjects had a significant reduction in fecal* Bifidobacterium* numbers with a trend towards increased* E. coli* [32]. In this study, we showed for the first time that the partial replacement of olive oil with fish oil significantly decreased numbers of* E. coli* and increased numbers of* Bifidobacterium* in stools and may tend to restore the bowel flora in rats under chronic ethanol feeding. However, the fecal microbiota is very complicated. It is important to improve the accuracy of the fecal microbiota analysis not only in quantitative technology but also in flora species. Comparison of the bacterial 16S ribosomal RNA gene sequence will be needed in our future studies.

At last, fish oil substitution has not been shown the dose-response relationship in ameliorating alcohol-induced liver damage according to the direct evidence of liver damage, that is, the histopathological analysis (Table 5). Only the score of fibrosis was significantly different between the EF25 and EF57 groups.

In this study, the olive oil was partially substituted by fish oil in the diet according to MUFA/PUFA ratios. The MUFA/PUFA ratio of 57% fish oil substitutions is 1.5 which is recommended in the human diet. Thus, we suggested that heavy drinkers should take care of the dietary fat sources, especially n-3 PUFA, for preventing the hepatic injury. If fish oil is considered to be a supplement, the dosage should be calculated according to the individual dietary patterns.

## 5. Conclusions

In conclusion, chronic ethanol feeding increased intestinal permeability, resulted in unbalanced fecal microbiota composition that may elevate plasma endotoxin levels, and consequently contributed to liver damage. However, substituting fish oil, especially provided in high dose, for olive oil under ethanol exposure normalizes the intestinal permeability and fecal microbiota composition, thus providing a low plasma endotoxin level and inflammatory responses, which exert ameliorative effects on ethanol-induced liver injuries in rats.

## Figures and Tables

**Table 1:** Composition of the experimental liquid diets in each group^1,2^.

Ingredients	C	CF25	CF57	E	EF25	EF57
	Concentration (g/L (1000 kcal))					
Casein	41.4	41.4	41.4	41.4	41.4	41.4
L-cysteine	0.5	0.5	0.5	0.5	0.5	0.5
DL-methionine	0.3	0.3	0.3	0.3	0.3	0.3
Corn oil	8.5	8.5	8.5	8.5	8.5	8.5
Olive oil	28.4	21.3	12.2	28.4	21.3	12.2
Safflower oil	2.7	2.7	2.7	2.7	2.7	2.7
Fish oil	0	7.1	16.2	0	7.1	16.2
Choline bitartrate	0.53	0.53	0.53	0.53	0.53	0.53
Fiber	10	10	10	10	10	10
Xanthan gum	4	4	4	4	4	4
ICN: AIN-76 vitamins	2.5	2.5	2.5	2.5	2.5	2.5
ICN: AIN-76 minerals	2.6	2.6	2.6	2.6	2.6	2.6
Maltodextrin	115.2	115.2	115.2	25.6	25.6	25.6
Ethanol	0	0	0	50	50	50

^1^C, control; CF25, control with 25% fish oil substituted for olive oil; CF57, control with 57% fish oil substituted for olive oil; E: ethanol; EF25, ethanol with 25% fish oil substituted for olive oil; EF57, ethanol with 57% fish oil substituted for olive oil.

^2^Casein, L-cysteine, DL-methionine, choline bitartrate, fiber, AIN-76 vitamins, AIN-76 minerals, and maltodextrin were purchased from ICN Biochemicals (Costa Mesa, CA, USA). Xanthan gum, ethanol, and glutamine were purchased from the Sigma-Aldrich (St. Louis, MO, USA). Corn oil and olive oil were purchased from the God Bene Enterprise (Yunlin, Taiwan). Safflower oil was purchased from the Taiwan Sugar Corporation (Taipei, Taiwan). Fish oil (VIVA Omega-3*™*) was provided by VIVA Life Science (Costa Mesa, CA, USA).

**Table 2:** The fatty acid compositions of the experimental diets^1,2,3^.

Fatty acid (mg)/39.6 g oil	C/E	CF25/EF25	CF57/EF57
Myristic acid (14:0)	8.2	7.9	7.4
Pentadecanoic acid (15:0)	0.0	0.0	0.0
Palmitic acid (16:0)	4668.0	3784.0	2651.0
Margaric acid (17:0)	14.0	12.2	9.9
Stearic acid (18:0)	1057.4	870.6	631.0
Nonadecanoic acid (19:0)	0.0	0.0	0.0
Arachidic acid (20:0)	165.9	135.4	96.3
Behenic acid (22:0)	69.4	59.0	45.6
Lignoceric acid (24:0)	35.1	30.2	23.9
Myristoleic acid (14:1)	0.0	0.0	0.0
Palmitoleic acid (16:1)	302.9	229.6	135.6
Oleic acid (18:1)	23323.7	18158.4	11538.2
Gadoleic acid (20:1)	131.0	110.9	85.2
Erucic acid (22:1)	2.0	2.0	2.0
Linoleic acid (18:2)	9358.2	8684.9	7821.9
Linolenic acid (18:3)	426.7	428.0	429.7
Octadecatetraenoic acid (18:4)	0.0	71.7	163.6
Arachidonic acid (20:4)	1.4	246.3	560.3
Eicosapentaenoic acid (20:5)	0.0	2185.4	4986.4
Heneicosapentaenoic acid (21:5)	0.0	93.0	212.2
Docosapentaenoic acid (22:5)	0.0	337.3	769.5
Docosahexaenoic acid (22:6)	0.0	1688.4	3852.4
Other fatty acids	9.2	9.2	9.2

Saturated fatty acids	6018.1	4899.3	3465.2
MUFAs	23759.6	18500.9	11760.9
PUFAs	9786.3	13734.9	18795.9
M/P	1/0.4	1/0.7	1/1.5

^1^C, control; CF25, control with 25% fish oil substituted for olive oil; CF57, control with 57% fish oil substituted for olive oil; E: ethanol; EF25, ethanol with 25% fish oil substituted for olive oil; EF57, ethanol with 57% fish oil substituted for olive oil.

^2^The contents of fatty acid are shown as the weight (mg) in 39.6 g oil of the liquid diets (the sum of corn oil, olive oil, safflower oil, and fish oil).

^3^These data are based on Taiwan Food and Drug Administration, Ministry of Health and Welfare-food nutrient database established by Taiwan Food Industry Research and Development Institute and National Pingtung University of Science and Technology, Taiwan.

**Table 3:** Effects of fish oil on the initial body weight (BW), final BW, liver weight, and relative liver weight in rats under chronic ethanol feeding^1,2,3^.

Group	Initial BW (g)	Final BW (g)	Liver weight (g)	Relative liver weight (%)
C	339.5 ± 4.6^a^	405.5 ± 5.5^c^	9.0 ± 0.2^a^	2.2 ± 0.1^a^
CF25	335.5 ± 3.7^a^	413.0 ± 5.9^c^	10.1 ± 0.2^bc^	2.4 ± 0.0^a^
CF57	339.0 ± 5.6^a^	419.8 ± 6.7^c^	10.2 ± 0.2^bc^	2.4 ± 0.1^a^
E	328.8 ± 3.0^a^	395.8 ± 7.1^b^	10.9 ± 0.5^c^	2.7 ± 0.1^b^
EF25	326.8 ± 6.7^a^	391.3 ± 8.8^b^	9.7 ± 0.4^ab^	2.8 ± 0.1^b^
EF57	332.7 ± 6.4^a^	394.2 ± 8.4^b^	10.6 ± 0.4^bc^	2.9 ± 0.1^b^

^1^Values are expressed as the mean ± SEM. Means in the same column with different superscript letters significantly differ (*p* < 0.05).

^2^C, control; CF25, control with 25% fish oil substituted for olive oil; CF57, control with 57% fish oil substituted for olive oil; E, ethanol; EF25, ethanol with 25% fish oil substituted for olive oil; EF57, ethanol with 57% fish oil substituted for olive oil.

^3^Relative liver weight: (liver weight/body weight) × 100%.

**Table 4:** Effects of fish oil on plasma aspartate transaminase (AST) and alanine transaminase (ALT) activities in rats under chronic ethanol feeding^1,2^.

Group	AST (U/L)	ALT (U/L)
C	85.0 ± 1.6^a^	53.2 ± 4.0^ab^
CF25	84.7 ± 2.5^a^	49.5 ± 3.7^a^
CF57	91.3 ± 3.4^ab^	50.0 ± 1.9^ab^
E	197.8 ± 22.5^d^	95.3 ± 15.7^c^
EF25	122.0 ± 3.3^bc^	76.2 ± 4.1^b^
EF57	142.2 ± 14.7^c^	71.2 ± 5.4^b^

^1^Values are expressed as the mean ± SEM. Means in the same column with different superscript letters significantly differ (*p* < 0.05).

^2^C, control; CF25, control with 25% fish oil substituted for olive oil; CF57, control with 57% fish oil substituted for olive oil; E, ethanol; EF25, ethanol with 25% fish oil substituted for olive oil; EF57, ethanol with 57% fish oil substituted for olive oil.

**Table 5:** Effects of fish oil on histopathological analysis of liver tissue in rats under chronic ethanol feeding^1,2^.

Group	Fatty change		Inflammatory cell infiltration	Degeneration and necrosis	Fibrosis
	Macrovesicular	Microvesicular			
C	0.0 ± 0.0^a^	0.0 ± 0.0^a^	0.0 ± 0.0^a^	0.0 ± 0.0^a^	0.0 ± 0.0^a^
CF25	0.3 ± 0.2^a^	0.2 ± 0.2^a^	0.6 ± 0.2^ab^	0.4 ± 0.2^ab^	0.0 ± 0.0^a^
CF57	0.5 ± 0.0^a^	0.6 ± 0.2^a^	0.6 ± 0.2^ab^	0.6 ± 0.2^ab^	0.4 ± 0.2^ab^
E	2.0 ± 0.3^d^	2.4 ± 0.2^c^	2.6 ± 0.2^e^	2.0 ± 0.0^d^	2.2 ± 0.5^d^
EF25	0.2 ± 0.2^ab^	1.6 ± 0.2^b^	1.4 ± 0.2^cd^	1.4 ± 0.2^cd^	1.6 ± 0.2^cd^
EF57	0.4 ± 0.4^abc^	1.4 ± 0.2^b^	1.8 ± 0.4^d^	1.0 ± 0.3^bc^	1.0 ± 0.0^bc^

^1^Values are expressed as the mean ± SEM. Means in the same column with different superscript letters significantly differ (*p* < 0.05).

^2^C, control; CF25, control with 25% fish oil substituted for olive oil; CF57, control with 57% fish oil substituted for olive oil; E, ethanol; EF25, ethanol with 25% fish oil substituted for olive oil; EF57, ethanol with 57% fish oil substituted for olive oil.

**Table 6:** Effects of fish oil on hepatic tumor necrosis factor- (TNF-) *α*, interleukin- (IL-) 1*β*, IL-6, and IL-10 levels in rats under chronic ethanol feeding^1,2^.

Group	TNF-*α* (pg/mg protein)	IL-1*β* (pg/mg protein)	IL-6 (pg/mg protein)	IL-10 (pg/mg protein)
C	60.4 ± 6.0^a^	62.6 ± 3.0^ab^	97.5 ± 5.7^a^	91.3 ± 3.4^a^
CF25	66.2 ± 5.1^b^	67.1 ± 3.0^b^	86.3 ± 7.6^a^	87.6 ± 5.8^a^
CF57	67.2 ± 3.2^b^	59.3 ± 1.3^ab^	91.5 ± 5.6^a^	81.4 ± 4.1^a^
E	89.0 ± 5.2^c^	76.5 ± 2.5^c^	118.1 ± 8.2^b^	118.0 ± 5.4^b^
EF25	59.5 ± 3.4^a^	56.8 ± 2.6^a^	94.0 ± 3.3^a^	88.5 ± 4.6^a^
EF57	75.2 ± 6.0^b^	65.0 ± 3.8^ab^	97.5 ± 5.9^a^	92.9 ± 4.6^a^

^1^Values are expressed as the mean ± SEM. Means in the same column with different superscript letters significantly differ (*p* < 0.05).

^2^C, control; CF25, control with 25% fish oil substituted for olive oil; CF57, control with 57% fish oil substituted for olive oil; E, ethanol; EF25, ethanol with 25% fish oil substituted for olive oil; EF57, ethanol with 57% fish oil substituted for olive oil.

**Table 7:** Effects of fish oil on endotoxin concentrations in rats under chronic ethanol feeding^1,2^.

Group	Endotoxin (EU/mL)
C	25.5 ± 2.6^b^
CF25	26.9 ± 0.3^b^
CF57	23.7 ± 1.7^a^
E	30.4 ± 1.8^c^
EF25	29.0 ± 3.6^c^
EF57	21.2 ± 1.6^a^

^1^Values are expressed as the mean ± SEM. Means in the same column with different superscript letters significantly differ (*p* < 0.05).

^2^C, control; CF25, control with 25% fish oil substituted for olive oil; CF57, control with 57% fish oil substituted for olive oil; E, ethanol; EF25, ethanol with 25% fish oil substituted for olive oil; EF57, ethanol with 57% fish oil substituted for olive oil.

**Table 8:** Effects of fish oil on the lactulose/mannitol (L/M) ratio in rats under chronic ethanol feeding^1,2^.

Group	L/M ratio
C	3.0 ± 0.0^b^
CF25	3.2 ± 0.0^b^
CF57	2.8 ± 0.1^a^
E	3.4 ± 0.2^c^
EF25	3.2 ± 0.2^b^
EF57	2.9 ± 0.0^ab^

^1^Values are expressed as the mean ± SEM. Means in the same column with different superscript letters significantly differ (*p* < 0.05).

^2^C, control; CF25, control with 25% fish oil substituted for olive oil; CF57, control with 57% fish oil substituted for olive oil; E, ethanol; EF25, ethanol with 25% fish oil substituted for olive oil; EF57, ethanol with 57% fish oil substituted for olive oil.

**Table 9:** Effects of fish oil on fecal microbiota composition in rats under long-term ethanol feeding^1,2,3^.

Group	Anaerobe (CFU/g)	*Escherichia coli* (CFU/g)	*Lactobacillus* (CFU/g)	*Bifidobacterium* (CFU/g)
C	7.4 ± 0.2^a^	6.1 ± 0.3^abc^	6.8 ± 0.2^a^	6.7 ± 0.1^c^
CF25	7.3 ± 0.1^a^	6.0 ± 0.2^ab^	6.9 ± 0.2^a^	6.4 ± 0.1^bc^
CF57	7.1 ± 0.1^a^	6.4 ± 0.1^bc^	6.7 ± 0.5^a^	6.5 ± 0.1^c^
E	7.4 ± 0.3^a^	6.7 ± 0.2^c^	6.2 ± 0.2^a^	6.0 ± 0.2^ab^
EF25	7.1 ± 0.2^a^	5.8 ± 0.3^a^	6.3 ± 0.4^a^	6.5 ± 0.2^c^
EF57	6.9 ± 0.2^a^	5.8 ± 0.1^ab^	6.5 ± 0.2^a^	6.6 ± 0.1^c^

^1^Values are expressed as the mean ± SEM. Means in the same column with different superscript letters significantly differ (*p* < 0.05).

^2^C, control; CF25, control with 25% fish oil substituted for olive oil; CF57, control with 57% fish oil substituted for olive oil; E, ethanol; EF25, ethanol with 25% fish oil substituted for olive oil; EF57, ethanol with 57% fish oil substituted for olive oil.

^3^CFU, colony forming units.
